# Determinants of acute malnutrition among 6–59 months old children in public hospitals in Gambella town, Southwest Ethiopia: unmatched case-control study

**DOI:** 10.3389/fnut.2023.1212504

**Published:** 2023-09-29

**Authors:** Abreha Addis Gesese, Luel Duoth Khot

**Affiliations:** ^1^Department of Clinical Nursing, Gambella Teachers Education and Health Science College, Gambella, Ethiopia; ^2^Department of Public Health Nutrition, School of Public Health, Mekelle University, Mekelle, Ethiopia

**Keywords:** determinants, acute malnutrition, 6–59 months old children, public hospitals, Gambella town, Southwest Ethiopia

## Abstract

**Background:**

Acute malnutrition is a severe public health issue caused by poor nutrition over a short period of time. It is a powerful predictor of mortality. The Gambella region’s risk factors for acute malnutrition, however, are not well understood. Thus, the risk factors for acute malnutrition were identified in this study.

**Methods:**

A facility-based unmatched-case control study design was conducted in public hospitals in Gambella town from February 15 to March 30, 2019. A total of 85 cases and 170 controls were included in the study. According to the average monthly caseload, children between the ages of 6 and 59 months were distributed among the public hospitals. Then, cases and controls were chosen using a systematic random sampling technique. A standardized, previously tested questionnaire was used to collect the data. EPI-data version 3.1 was used to enter the data, which was subsequently exported to SPSS version 20 for analysis. Statistical significance was set at p 0.05 for the bivariate and multivariable logistic regressions that were employed.

**Results:**

Household income of <=1,500 birr [AOR = 2.46 at 95% CI (1.37–4.39)], being unemployed [AOR = 2.37 at 95% CI (1.34–4.20)], rural residence [AOR = 1.96 at 95% CI (1.10–3.51)], having diarrhea [AOR = 2.47, 95% CI (1.36–4.51)], fever [AOR = 2.05, 95% CI (1.03–4.10)], and pneumonia (AOR = 2.41), and exclusive breast feeding (AOR = 1.96, and exclusive breast feeding [AOR = 1.96, 95% CI (1.18–4.91)], and exclusive breast feeding (AOR = 1.96) minimum dietary diversity [AOR = 2.86, 95% CI (1.06–3.64)], minimum dietary diversity [AOR = 2.86, 95% CI (1.37–5.95)], weight measurement at birth [AOR = 0.86, 95% CI (0.08–0.93)], unplanned birth of the child [AOR = 1.97, 95% CI (1.02–3.78)], and non-immunized [AOR = 4.12, 95% CI (1.05–16.13)] were associated with acute malnutrition.

**Conclusion:**

This research identified multiple risk factors for acute malnutrition in children aged 6–59 months, suggesting health interventions, and programs at all levels of the system executed in an organized manner with substantial program ramifications.

## 1. Background

Acute malnutrition is a recent and significant weight loss (wasting) brought on by an acute food deficit and/or sickness and its evaluated by Weight for height or the Mid-Upper Arm Circumference (MUAC) (1). Acute malnutrition includes both moderate acute malnutrition (MAM) and severe acute malnutrition (SAM) (2). Political unrest, sluggish economic growth, and lack of education are a few of the more fundamental issues; frequent illnesses and poor diet are some of the underlying causes and risk factors contribute to undernutrition in children (3, 4).

Child undernutrition continues to be a serious public health issue which is the main cause of child morbidity and mortality worldwide (5, 6). Children with SAM have a nine-fold higher chance of dying (7), 35% of the illness burden and 11% of all DALYs globally (5, 7). Around 55 million children under the age of five are wasted globally, with South-central Asia reporting the highest estimate (16%). Of this, 3.5% are seriously wasted with the highest concentration in south-central and middle Africa (8). About 80% of (36–41 million) children with acute malnutrition reside in nations with low incomes (9, 10), one in every three children in Sub-Saharan Africa (SSA) with socioeconomic inequality (11, 12). Many children experience the symptoms of acute malnutrition (13–17). In Ethiopia, severe and moderate malnutrition are also responsible for 51% of under-five child mortality, with moderate malnutrition accounting for 40% of these deaths (18).

Poverty, parental illiteracy, parental decision-making, insufficient feeding practices, large family size, non-exclusive breastfeeding, diarrhea, low birth weight, immunization status, disturbed (broken) family, maternal hand-washing habit, and repeated pregnancies are risk factors for acute malnutrition in children. Through its direct, non-synergistic effects on the mortality from infectious diseases, it also occurs with short-term factors like seasonal variation in food availability, acute food shortages, changes in social or economic policies, and the occurrence of illnesses (4–6, 19–21).

Ethiopia’s economic growth and transformation plan has brought a decrease in stunting and the percentage of underweight children. However, acute malnutrition remained largely constant and persisted as a significant public health issue with no significant change between 11% in 2005 and 10% in 2016. The fourth Health Sector Development Program (HSDP-IV), which was launched by the Ethiopian government under the Growth and Transformation Plan (GTP), gave more consideration to nutrition than the three earlier plans in order to reduce the prevalence of acute malnutrition from 11 to 3% (22). Meanwhile, compared to the national average of 10, 14.1, and 1.6% of Gambella children were severely wasted. This shows that the issue’s magnitude is still significant and that it will continue to be difficult to reduce child mortality (23). In each hospital, more than 100 children under the age of five are typically admitted each month as referral patients from host communities and refugees from the displaced settings of South Sudan. Gambella Town has 85% coverage for health services (24).

Few studies conducted in Ethiopia showed inconsistent findings and the majorities were cross-sectional studies which did not adequately address the risk factors for acute malnutrition (21, 23, 25–30). This study makes unique because it is conducted in a setting where large number of refugees reside and get services besides the host communities. It also incorporates different dimensions of factors of acute malnutrition. This study will aid in the design and prioritization of high-impact nutrition interventions for effective management, at the health institutions and community level by health care providers, including health extension workers and NGOs who work in outpatient and established nutrition programs. The regional health bureau will also be benefitted in preventing the onset of acute malnutrition and its consequences among vulnerable age groups. Overall, the findings will have significant contribution for the improvement of programs and policies on child nutritional status, food security in the impacted neighborhoods, and other demographic subgroups such as refugees. Finally, this finding will help as a baseline data for researchers and scholars.

## 2. Methods

### 2.1. Study design, setting, and period

In the public hospitals of Gambella town, a facility-based unmatched case–control study design was carried out from February 15 to March 30, 2019. It is situated 768 kilometers from Addis Ababa, the country’s capital city, in the Gambella area in southwest Ethiopia. The area is divided into three zones, 13 woredas, and one unique woreda called Itang, which has a variety of ethnic groups and Amharic as its official working language. The region has a total population of 306,916 people, according to the 2007 National Census. The town has roughly 55,394 residents in total (22). According to the population forecast for the 2011 Ethiopian Fiscal Year (EFY), the town has 8,473 children under the age of five, of which 95% (8027) were between the ages of six and 59 months of age. There were 30 private clinics, 55 health facilities, 66 health posts, and five functioning hospitals in the area. The study was conducted in these two hospitals since there are 30 different private clinics and one general hospital in Gambella town, according to the 2016 report from the town’s administrative health office (24).

### 2.2. Source and study population

The source population consisted of all 6-59-month-old children who attended the public hospitals in Gambella town during the study period. The study population consisted of all randomly chosen kids between the ages of 6 and 59 months. Participants in the study are divided into two groups: cases and controls. According to WHO’s 2006 New Growth Standard, cases included children with MUAC >11.5 cm and 12.5 cm (MAM) and 11.5 cm (SAM) with acute malnutrition and either presence or absence of bilateral pitting edema of nutritional origin. Children who had MUAC 12.5 cm or who did not have acute nutritional deficiency but did have another medical issue served as controls.

### 2.3. Inclusion and exclusion criteria

The cases were children admitted to a pediatric ward or under-five clinic with MUAC <12.5 cm with or without edema according to WHO 2006 new growth standard (31). Children admitted to the pediatric ward or visiting the well-baby clinic with no acute malnutrition and with MUAC≥12.5 cm were considered as controls (31). All admissions to the inpatient (IPW) and outpatient pediatric wards (POD), under-five clinics, or well-baby clinics during the study period were included in the study. However, children with physical deformities, unconscious, and burned hands, which make anthropometric measurements inconvenient were excluded from the study for both Cases and controls.

### 2.4. Study variables

#### 2.4.1. Dependent variable

Is acute malnutrition (SAM or MAM). SAM = MUAC <11.5 cm or MUAC >11.5 cm and <12.5 cm for (MAM), and either presence or absence of bilateral pitting edema in under five children. Children with weight-for-height Z-score (WHZ) < −3 SD from the median value of WHO’s 2006 reference data were considered as wasted (acutely malnourished).

#### 2.4.2. Independent variables

Socio-demographic and economic status variables: (Family size, age, sex, occupation, and parental education, ethnicity, religion and place of residence, monthly income); Maternal characteristics (age, number of under-five children) and Child characteristics and caring practices (sex, age, immunization status, hygiene); Nutrition-related variable (feeding practices, DDS, Household food security); Health-related characteristics (health care seeking, and morbidity status of previous illness); and community factors (distance and sanitation of water supply) were considered as.

### 2.5. Sample size determination

The sample size was determined using Epi info version 7software using two population proportions formula with the following assumptions: 95% CI, 80% power, and a ratio of 1:2 (case: controls), using a study from Nekemte, Oromia region reported that the expected frequency of family size was 42.9% for wasting (2.42 odd ratios) (32). Thus, considering the 10% non-response rate, the final sample size was 255 (85 cases and 170 controls).

### 2.6. Sampling technique and procedure

**Figure 1:**
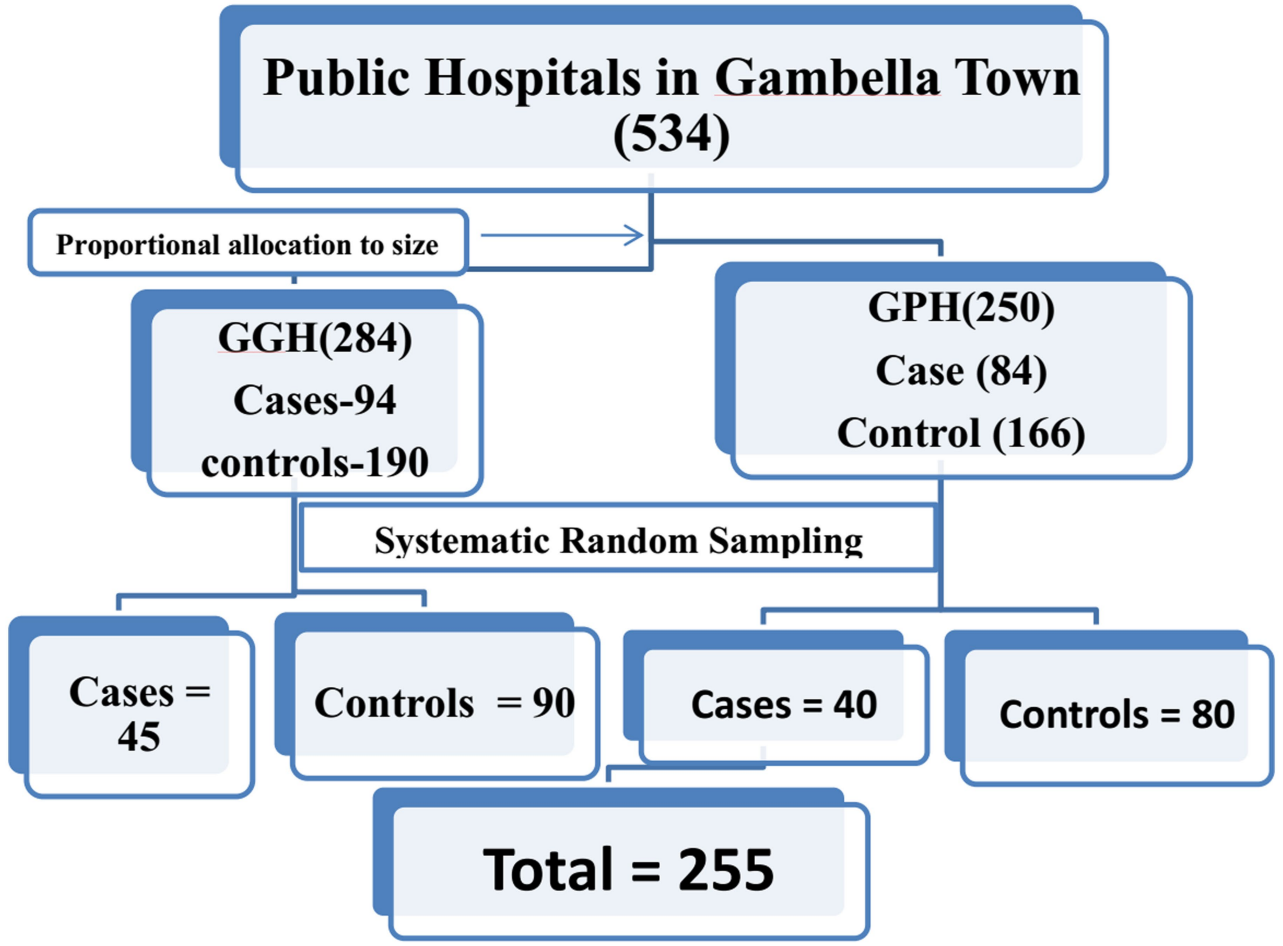
Sampling technique on risk factors of acute malnutrition.

The town’s general and primary hospitals were both considered for the study. First, the sample was proportionally distributed among the hospitals based on the monthly average for cases involving children between the ages of 6 and 59 months. Then, a systematic random sampling technique was used to choose 45 cases and 90 controls at every second child from 94 cases and 190 controls from the Gambella General Hospital, using the registration book as a sampling frame. The registration book was used as a sampling frame in the primary hospital, where 40 cases and 80 controls were chosen from 84 cases and 166 controls using a systematic random sampling procedure at every second kid (24). The following mothers or caregivers who consented to participate and met the inclusion criteria were recruited at every other second mother or caregiver after a mother or caregiver of the child declined to participate in the study. Ultimately, 170 controls and 85 cases that visited or were admitted to these hospitals were chosen (Figure 1).

### 2.7. Data collection technique and procedure

Data were gathered using a standardized pretested questionnaire that was created after analyzing several types of literature from related studies (3, 9, 32–37). To ensure the questionnaire’s clarity and consistency, it was first prepared in English, translated into Amharic, and then translated back into English. The instrument includes sociodemographic and economic factors, mother traits, child traits, and parenting behaviors, nutrition-related factors, and health-related traits. After then, the questionnaire was given out in Amharic. Six diploma health professionals with a minimum of 1 year of work experience, two Bsc nurse supervisors, and the primary investigator all participated in the data collection. The armband/tape was used to screen the children with MUAC 12.5 cm cases and MUAC 12.5 cm controls per WHO new growths standard, and it was used to measure MUAC. We used the FAO and FANTA recommendations to gauge children’s eating habits such EBF, FFQ, and minimum recommended food intake.

### 2.8. Data quality control

Pre-test was done on 5% of the total sample size in the Abol health center of a comparable demographic area to ensure the validity of the data. Following that, the appropriate modifications were made. The lead investigator provided 2 days of training to data collectors and supervisors on the study tools, consent form, how to conduct interviews, how to collect data on anthropometric measurements, and other topics. The collected data was then carefully examined and confirmed for completeness and relevancy by the supervisors and investigators.

### 2.9. Data processing and analysis

Each survey that was gathered in the field was manually cleaned up after being reviewed for accuracy, missing values, and odd responses. The obtained data were then coded, entered, and verified as complete using the statistical tool EPI-data version 3.1, and exported to SPSS version 20 for data analysis. Then, categorical variables were described using descriptive statistics like frequencies and percentages. Parametric continuous variables were described using the mean and standard deviation. The logistic regression analysis with two and more variables is performed to find the risk factors for acute malnutrition. In the bivariate analysis, candidate predictors for the multivariable analysis were chosen using a *p*-value of <=0.25. The final model’s variables with *p*-values under 0.05 were deemed statistically significant risk factors of acute malnutrition. Hosmer-Lemeshow was used to evaluate the model’s fitness at p 0.05.

### 2.10. Operational definition

#### 2.10.1. Case

Child with MUAC >11.5 cm and <12.5 cm (MAM) and <11.5 cm (SAM) with acute malnutrition either presence or absence of bilateral pitting edema of nutritional origin according to WHO 2006 new growth standard including weight-for-height Z-score (WHZ) < −3 SD (31).

#### 2.10.2. Control

A child with MUAC ≥12.5 cm or without any of the above definitions of acute malnutrition of nutritional origin but has other medical problem (31).

## 3. Results

### 3.1. Socio-demographics and economic characteristics of study participants

A total of 255 mothers (85 cases and 170 controls) were questioned, yielding a 100% response rate. More than one-third of the participants in the study were female-headed families, with 34 (40.0%) cases and 55 (32.4%) controls. Males made up approximately 44 (51.8%) and 78 (45.9%) of the children in the cases and controls, respectively. The majority of child’s age was between 12 and 23 months of age. Family sizes of five or more were common in about three-quarters of the cases, 66 (77.6%) and 148 (87.1%) of the controls. The mean household/family income (SD) for the cases and controls, respectively, was 1562.18 (1347.04) and 3569.74 (2686.54) birr per month and the majority had <=1,500 Ethiopian birr (Table 1).

**Table 1:** Socio-demographic and economic characteristics of the study participants in public hospitals of Gambella town, Southwest Ethiopia 2019 (*n* = 255).

Variables	Category	Cases		Controls	
		Frequency	Percentage	Frequency	Percentage
Sex of head of household	Male	51	60.0	115	67.6
	Female	34	40.0	55	32.4
Child’s sex	Male	44	51.8	78	45.9
	Female	41	48.2	92	54.1
Child’s age	6–11 months	24	28.2	34	20.0
	12–23 months	33	38.8	99	58.2
	24–59 months	28	32.9	37	21.8
Mother’s age	≤34	45	52.9	106	62.4
	≥35	40	47.1	64	37.6
Ethnicity	Agnuak/Komo	22	25.9	41	24.1
	Nuer/Opo	33	38.8	73	42.9
	Mejagn/others	30	35.3	56	32.9
Religion	Orthodox	23	27.1	44	25.9
	Protestants	46	54.1	103	60.6
	Others	16	18.8	23	13.5
Residence	Urban	30	35.3	95	55.9
	Rural	55	64.7	75	44.1
Marital status	Married	57	67.1	118	69.4
	Divorced/widowed/single	28	32.9	52	30.6
Family size	<5	19	22.4	22	12.9
	≥5	66	77.6	148	87.1
Number of children 6–59 months in the household	<3	55	64.7	108	63.5
	≥3	30	35.3	62	36.5
Maternal education	Illiterate	58	68.2	84	49.4
	Literate	27	31.8	86	50.6
Paternal education	Literate	44	51.8	82	48.2
	Illiterate	41	48.2	88	51.8
Occupation of the mothers	Unemployed	54	63.5	75	44.1
	Government employee/NGO	31	36.5	95	55.9
Household income	≤1,500 birr	58	68.2	77	45.3
	>1,500 birr	27	31.8	93	54.7
Who decides on the use of the money earned	Mainly husband	67	78.8	143	84.1
	Both jointly	18	21.2	27	15.9
Maternal autonomy in decision making	Yes	42	49.4	91	53.5
	No	43	50.6	79	46.5
Age at first birth in years	15–24	60	70.6	111	65.3
	≥25	25	29.4	59	34.7
Age at last birth in years	≤34	33	38.8	40	23.5
	≥35	52	61.2	130	76.5
Total number of children ever born	<5	39	45.9	105	61.8
	≥5	46	54.1	65	38.2

### 3.2. Hygiene and sanitation characteristics

The majority of the mothers in the two groups 47 (55.3%) of the cases and 78 (45.9%) of the control households got their water from rivers or wells. 49 (57.2%) of cases and 148 (87.1%) of controls had average daily household water consumptions of > = 50 l, with the majority of cases and controls average took > = 30 min to get water from the source reaching. Nearly two-thirds of the households 53 (62.4%) of the cases and 97 (57.1%) of the controls treat water straining by cloth or use it as it is at home (Table 2).

**Table 2:** Hygiene and sanitation characteristic of the study participants in public hospitals of Gambella town, Southwest Ethiopia 2019 (*n* = 255).

Variables	Category	Cases *N* (%)	Controls *N* (%)
Household main source of water	River/well	47 (55.3)	78 (45.9)
	Public/private tape	38 (44.7)	92 (54.1)
Critical times of hand washing	Before/after latrine use	25 (29.4)	53 (31.2)
	After cleaning child faeces	29 (34.1)	49 (28.8)
	Before preparing/serving food	31 (36.5)	68 (40.0)
Means of hand washing	Using water/ash	44 (51.8)	69 (40.6)
	Using soap always/sometimes	41 (48.2)	101 (59.4)
Daily *per capita* household consumption of water	<50 l	36 (42.4)	22 (12.9)
	≥50 l	49 (57.6)	148 (87.1)
Time taken to fetch water	<30 min	39 (45.9)	44 (25.9)
	≥30 min	46 (54.1)	126 (74.1)
Methods of water treatment	Boiling/adding chlorines	32 (37.6)	73 (42.9)
	Straining by cloth/used as it is	53 (62.4)	97 (57.1)
Availability of household latrine	Yes	42 (49.4)	107 (62.9)
	No	43 (50.6)	63 (37.1)
Waste disposal	Open field disposal/burning	31 (36.5)	43 (25.3)
	In a common/private hole/in a pit	54 (63.5)	127 (74.7)

### 3.3. Feeding practices of the children

In the 24 h before visiting a medical facility, mothers of cases made up 58 (68.2%) and controls made up 53 (31.2%) of the mothers who gave pre-lacteal food or fluid to their children. After an hour during delivery, 55 (64.7%) of the cases and 76 (44.7%) of the controls started breastfeeding. About 126 (74.1%) of the controls and 44 (51.8%) of the cases had exclusively breastfed their babies (Table 3).

**Table 3:** Infant and young child feeding practice of the study participants in public hospitals of Gambella town, Southwest Ethiopia 2019 (*n* = 255).

Status of IYCF	Category	Case, *N* (%)	Control, *N* (%)
Prelacteal feeds	Yes	58 (68.2)	53 (31.2)
	No	27 (31.8)	117 (68.8)
Timely initiation of breast feeding	Within one hour	30 (35.3)	94 (55.3)
	After one hour	55 (64.7)	76 (44.7)
Exclusively breast feeding	Yes	44 (51.8)	126 (74.1)
	No	41 (48.2)	44 (25.9)
Continued breast feeding	Yes	44 (51.8)	105 (61.8)
	No	41 (48.2)	65 (38.2)
Bottle feeding of children	Yes	56 (65.9)	71 (41.8)
	No	29 (34.1)	99 (58.2)
Timely introduction of solid, semi-solid complementary foods	<6 month	60 (70.6)	52 (30.6)
	≥6 month	25 (29.4)	118 (69.4)
Minimum meal frequency	Yes	42 (49.4)	64 (37.6)
	No	43 (50.6)	106 (62.4)
Minimum acceptable diet	In adequate	66 (77.6)	91 (53.5)
	Adequate	19 (22.4)	79 (46.5)

### 3.4. Child and maternal health characteristics

In the cases and controls of the study participants, 44 (51.8%) and 71 (41.8%), respectively, of the children were born at home. Similar to the mothers of cases, 43 (50.6%) and around 47 (27.6%) of the mothers of controls, respectively, had never given their kids any vaccinations. Two weeks prior to the research period, approximately 55 (64.7%) of the children from cases and over one-third 63 (37.1%) of the children from controls had diarrhea. Consistent with febrile illness, 58 (68.2%) of the cases’ children and 67 (39.4%) of the controls’ children reported having a fever in the 2 weeks just before the study’s start.

Regarding maternal health traits, approximately 35 (41.2%) of mothers in cases and 47 (27.6%) of mothers in controls had pre-pregnancy weights of less than 45 kg in their most recent pregnancies, respectively. 54 (31.8%) and 44 (51.8%) of mothers of controls and cases, respectively, had never gone to a medical facility for ANC follow-up during the pregnancy. Pertaining to mothers’ awareness of family planning Mothers from both case and control groups, 50 (58.8%) and 71 (41.8%), respectively, had no knowledge of family planning (Table 4).

**Table 4:** Child and maternal health characteristics of the study in public hospitals of Gambella town, Southwest Ethiopia 2019 (*n* = 255).

Variable	Category	Case (%)	Control (%)
Child birth order	≤3	42 (49.4)	102 (60.0)
	≥4	43 (50.6)	68 (40.0)
Place of delivery	Home	44 (51.8)	71 (41.8)
	Health institution	41 (48.2)	99 (58.2)
Gestational age at birth	<9 months	48 (56.5)	78 (45.9)
	≥9 months	37 (43.5)	92 (54.1)
Weight measurement at birth	Yes	47 (55.3)	116 (68.2)
	No	38 (44.7)	54 (31.8)
Birth types	Single	71 (83.5)	138 (81.2)
	Multiple/twins	14 (16.5)	32 (18.8)
Planned birth of the child	Yes	54 (63.5)	140 (82.4)
	No	31 (36.5)	30 (17.6)
Ever immunized	Yes	42 (49.4)	123 (72.4)
	No	43 (50.6)	47 (27.6)
Vitamin A supplementation	Yes	42 (49.4)	119 (70.0)
	No	43 (50.6)	51 (30.0)
Child had diarrhea in the last 2 weeks	Yes	55 (64.7)	63 (37.1)
	No	30 (35.3)	107 (62.9)
Child had fever in the last 2 weeks	Yes	58 (68.2)	67 (39.4)
	No	27 (31.8)	103 (60.6)
Child had respiratory disease in the last 2 weeks	Yes	57 (67.1)	65 (38.2)
	No	28 (32.9)	105 (61.8)
Pre-pregnancy weight in the last pregnancy	<45 kg	35 (41.2)	47 (27.6)
	≥45 kg	50 (58.8)	123 (72.4)
Consumption of extra meal during pregnancy or lactation	Yes	41 (48.2)	115 (67.6)
	No	44 (51.8)	55 (32.4)
Health status during pregnancy	Good	47 (55.3)	115 (67.6)
	Not good/sick	38 (44.7)	55 (32.4)
ANC follow up	Yes	41 (48.2)	116 (68.2)
	No	44 (51.8)	54 (31.8)
Family planning knowledge	Yes	35 (41.2)	99 (58.2)
	No	50 (58.8)	71 (41.8)

### 3.5. Dietary diversity

Foods from cereals (grains, roots, and tubers) were consumed by about 38 (44.7%) cases and 91 (53.5%) controls of research participants, respectively. Foods from legumes and nuts were consumed by half of 43 (50.6%) of the cases and 98 (57.6%) of the controls, respectively. 51 (60.0%) of the cases and 92 (54.1%) of the controls consumed more than half of their calories from fruits and vegetables high in vitamin A. Similarly, over two-thirds 111 (65.3%) of the controls and half of 43 (50.6%) of the cases consumed foods made of flesh (meat, fish, poultry, and organ meat) (Table 5).

**Table 5:** Dietary diversity score of the study participants in public hospitals of Gambella town, Southwest Ethiopia 2019 (*n* = 255).

Food groups	Case (%)		Control(%)	
	Yes	No	Yes	No
Cereals (grains, roots, and tubers)	38 (44.7)	47 (55.3)	91 (53.5)	79 (46.5)
Legumes and nuts	43 (50.6)	42 (49.4)	98 (57.6)	72 (42.4)
Vitamin A rich fruits and vegetables	51 (60.0)	34 (40.0)	92 (54.1)	78 (45.9)
Others fruits and vegetables	41 (48.2)	44 (51.8)	89 (52.4)	81 (47.6)
Flesh food (meat, fish, poultry and liver/organ meat)	43 (50.6)	42 (49.4)	111 (65.3)	59 (34.7)
Eggs	51 (60.0)	34 (40.0)	121 (71.2)	49 (28.8)
Dairy products	42 (49.4)	43 (50.6)	118 (69.4)	52 (30.6)
Minimum dietary diversity	50 (58.8)	35 (41.2)	136 (80.0)	34 (20.0)

### 3.6. Food insecurity access scale measurement tool (HFIAS)

In the previous 4 weeks, 75 (44.1%) and 52 (61.2%) of the household members from the cases and controls, respectively, expressed concern that their home would not have enough food. A little over two-thirds, or 57 (67.1%) of the household members in the cases and 70 (41.2%) of the household members in the controls reported being unable to eat the types of foods they preferred and, similarly, any household members had consumed a little variety of foods as a result of a lack of resources. In the last 4 weeks, 40 (47.1%) cases and 68 (40.0%) controls were affected (Table 6).

**Table 6:** Household food insecurity access scale of the study participants in public hospitals of Gambella town, Southwest Ethiopia 2019 (*n* = 255).

Variables	Category	Cases *N* (%)	Controls *N* (%)
In the past 4 weeks, did you worry that your household would have not enough food	Yes	52 (61.2)	75 (44.1)
	No	33 (38.8)	95 (55.9)
In the past 4 weeks, were you/any household member not able to eat the kind of food you preferred b/c of a lack of resources	Yes	56 (65.9)	70 (41.2)
	No	29 (34.1)	100 (58.8)
In the past 4 weeks, did you/any household members have to eat a limited variety of food due to a lack of resources	Yes	40 (47.1)	68 (40.0)
	No	45 (52.9)	102 (60.0)
In the past 4 weeks, did you/any household members have to eat a some foods that you really did not want to eat b/c of a lack of resources to obtained others types of food	Yes	39 (45.9)	65 (38.2)
	No	46 (54.1)	105 (61.8)
In the past 4 weeks, was there ever no food to eat of any kind in your household because of lack of resources to get food?	Yes	36 (42.4)	71 (41.8)
	No	49 (57.6)	99 (58.2)
In the past 4 weeks, did you or any household member go to sleep at night hungry because there was not enough food?	Yes	34 (40.0)	69 (40.6)
	No	51 (60.0)	101 (59.4)
In the past 4 weeks, did you or any household member go a whole day and night without eating anything because there was not enough food?	Yes	42 (49.4)	74 (43.5)
	No	43 (50.6)	96 (56.5)
In the past 4 weeks, did you or any household member eats a smaller meal than you felt you needed because there was not enough food?	Yes	35 (41.2)	64 (37.6)
	No	50 (58.8)	106 (62.4)
In the past 4 weeks, did you or any other household member eat fewer meals in a day because there was not enough food?	Yes	41 (48.2)	73 (42.9)
	No	44 (51.8)	97 (57.1)

### 3.7. Anthropometric profiles of the children

More than half, or 52 (61.2%) of families from cases and 75 (44.1%) from controls, reported having worried about running out of food in the previous 4 weeks, respectively. Almost two-thirds, or 57 (67.1%) of the household members from the cases and 70 (41.2%) from the controls, reported being unable to eat the types of foods they preferred due to a lack of resources. In a similar vein, any household members who had eaten a small variety of foods as a result of a lack of resources also reported experiencing this. In the last 4 weeks, there were 40 (47.1%) cases and 68 (40.0%) controls.

### 3.8. Risk factors of acute malnutrition

A significant correlation was found between acute malnutrition and household income of <=1,500 birr [AOR = 2.46 at 95% CI (1.37–4.39)], being unemployed [AOR = 2.37 at 95% CI (1.34–4.20)], child age [AOR = 0.44 at 95% CI (0.23–0.83)], the total number of children ever born [AOR = 1.78 at 95% CI (1.02–3.09)], daily *per capita* household consumption of fewer than 50 liters [AOR = 3.86 at 95% CI (1.88–7.94)], method of water treatment [AOR = 0.56 at 95% CI (0.32–0.98)] and availability of household latrine [AOR =1.74 at 95% CI (1.03–2.95)], non-exclusive breast feeding(EBF) [AOR = 1.96, 95% CI (1.06–3.64)], minimum dietary diversity [AOR = 2.86, 95% CI (1.37–5.95)], weight measurement at birth [AOR = 0.26, 95% CI (0.08–0.93)], unplanned birth of the child [AOR = 1.97, 95% CI (1.02–3.78)], ever immunized [AOR = 4.12, 95% CI (1.05–16.13)], childhood illnesses such as diarrhea [AOR = 2.47, 95% CI (1.36–4.51)], fever [AOR =2.05, 95% CI (1.03–4.10)] and pneumonia [AOR =2.41, 95% CI (1.18–4.91)]. Similarly, pre-pregnancy weight in the last pregnancy [AOR = 1.93, 95% CI (1.10–3.38)], absence of ANC follow up [AOR =2.39, 95% CI (1.39–4.11)], consumption of dairy products [AOR = 0.34, 95% CI (0.16–0.72)], inadequate intake of other fruits and vegetables (AOR = 2.59, 95% CI (1.33–5.06)) and inability to eat preferred food due to lack of resources [AOR =3.20, 95% CI (1.46–7.03)] (Table 7).

**Table 7:** Risk factors of acute malnutrition among of the study participants in public hospitals of Gambella town, Southwest Ethiopia 2019 (*n* = 255).

Variables	Categories	Cases *N* (%)	Controls *N* (%)	COR, 95%CI	AOR, 95%CI
Household income	≤1,500 birr	58 (68.2)	77 (45.3)	2.60 (1.50–4.49)*	2.46 (1.37–4.39)**
	>1,500 birr	27 (31.8)	93 (54.7)	1.00	1.00
Occupation of the mother	Unemployed	54 (63.5)	75 (44.1)	2.21 (1.29–3.77)*	2.37 (1.34–4.20)**
	Government employee/NGO	31 (36.5)	95 (55.9)	1.00	1.00
Resident	Urban	30 (35.3)	95 (55.9)	1.00	1.00
	Rural	55 (64.7)	75 (44.1)	2.32 (1.36–3.98)*	1.96 (1.10–3.51)**
Child age	6–11 months	24 (28.2)	34 (20.0)	0.93 (0.46–1.91)	
	12–23 months	33 (38.8)	99 (58.2)	0.44 (0.24–0.83)*	0.44 (0.23–0.83)**
	24–59 months	28 (33.0)	37 (21.8)	1.00	1.00
Total number of children ever born	<5	39 (45.9)	105 (61.8)	1.00	1.00
	≥5	46 (54.1)	65 (38.2)	1.91 (1.13–3.23)*	1.78 (1.02–3.09)**
Daily water consumption	<50 l	36 (42.4)	22 (12.9)	4.94 (2.66–9.20)*	3.86 (1.88–7.94)**
	≥50 l	49 (57.6)	148 (87.1)	1.00	1.00
Methods of water treatment	Boiling/adding chlorines	32 (37.6)	73 (42.9)	1.00	1.00
	Straining by cloth/used as it is	53 (62.4)	97 (57.1)	1.25 (0.27–0.78)*	0.56 (0.32–0.98)**
Availability of household latrine	Yes	42 (49.4)	107 (62.9)	1.00	1.00
	No	43 (50.6)	63 (37.1)	1.74 (1.03–2.95)*	1.74 (1.03–2.95)**
Exclusively breast feeding	Yes	44 (51.8)	126 (74.1)	1.00	1.00
	No	41 (48.2)	44 (25.9)	2.67 (1.55–4.61)*	1.96 (1.06–3.64)**
Minimum dietary diversity	Yes	50 (58.8)	136 (80.0)	1.00	1.00
	No	35 (41.2)	34 (20.0)	2.80 (1.58–4.96)*	2.86 (1.37–5.95)**
Weight measurement at birth	Yes	47 (55.3)	116 (68.2)	1.00	1.00
	No	38 (44.7)	54 (31.8)	1.74 (1.02–2.97)*	0.26 (0.08–0.93)**
Planned birth of the child	Yes	54 (63.5)	140 (82.4)	1.00	1.00
	No	31 (36.5)	30 (17.6)	2.68 (1.48–4.84)*	1.97 (1.02–3.78)**
Ever immunized	Yes	42 (49.4)	123 (72.4)	1.00	1.00
	No	43 (50.6)	47 (27.6)	2.68 (1.56–4.61)*	4.12 (1.05–16.13)**
Child had diarrhea	Yes	55 (64.7)	63 (37.1)	3.11 (1.81–5.36)*	2.47 (1.36–4.51)**
	No	30 (35.3)	107 (62.9)	1.00	1.00
Child had fever	Yes	58 (68.2)	67 (39.4)	3.30 (1.90–5.73)*	2.05 (1.03–4.10)**
	No	27 (31.8)	103 (60.6)	1.00	1.00
Child had Pneumonia	Yes	57 (67.1)	65 (38.2)	3.29 (1.90–5.69)*	2.41 (1.18–4.91)**
	No	28 (32.9)	105 (61.8)	1.00	1.00
Pre-pregnancy weight in the last pregnancy	<45 kg	35 (41.2)	47 (27.6)	1.83 (1.06–3.17)*	1.93 (1.10–3.38)**
	≥ 45 kg	50 (58.8)	123 (72.4)	1.00	1.00
ANC follow up	Yes	41 (48.2)	116 (68.2)	1.00	1.00
	No	44 (51.8)	54 (31.8)	2.31 (1.35–3.93)*	2.39 (1.39–4.11)**
Other fruits and vegetables	Yes	41 (48.2)	89 (52.4)	1.00	1.00
	No	44 (51.8)	81 (47.6)	1.18 (1.34–4.71)*	2.59 (1.33–5.06)**
Dairy products	Yes	42 (49.4)	118 (69.4)	1.00	1.00
	No	43 (50.6)	52 (30.6)	2.32 (0.22–0.87)*	0.34 (0.16–0.72)**
Unable to eat the preferred food	Yes	57 (65.9)	70 (41.2)	2.91 (1.69–5.02)*	3.20 (1.46–7.03)**
	No	28 (34.1)	100 (58.8)	1.00	1.00

*indicates COR and **indicates AOR value at *p* < 0.05.

## 4. Discussion

This case control study assessed determinants of acute malnutrition among children aged 6–59 months in public hospitals in Gambella Town, Southwest Ethiopia. This study identified multiple risk factors for acute malnutrition. As a result, maternal occupation, household income, and place of residence have demonstrated a substantial connection with acute malnutrition among socio-demographic and economic factors (9, 37, 38). However, family size was not a factor in our study, despite the fact that several studies (9, 32, 37) have linked it to an increased risk of acute malnutrition due to overcrowding and inadequate spacing. This discrepancy may be brought about by differences in the socioeconomic level of the study location, study design, and sample size.

Compared to children of working mothers, children of unemployed mothers were 2.4 times more likely to have acute malnutrition. This finding is consistent with a research from the southern Ethiopian region of Kemba woreda (35). This may be a result of the time-consuming activities unemployed mothers engage in due to their hard employment with limited the amount of time available for child care (35). However, studies from Shashogo woreda, Ethiopia (36) and Ghana (3) found no link between maternal occupation and acute malnutrition.

Childhood diseases were found to be at substantial risk of acute malnutrition. This is supported by studies conducted in different settings of Ethiopia and Kenya children that child with diarrhea were more likely to experience acute malnutrition (32, 36, 39, 40). Likewise, children who had fevers or other febrile illnesses were also found to have acute malnutrition, which shows consistent with studies conducted elsewhere (32, 34, 36, 39). Similar to studies from Gaza, Palestine, and the urban slum of Nagpur, India, the presence of respiratory infections in the 2 weeks prior to the data collecting period was also discovered to be a significant risk factor for child acute malnutrition (39, 40).

Youngsters who were not immunized had a four-fold greater chance of developing acute malnutrition than youngsters who had received the recommended immunizations. Similar results were observed from Machakel and Shinile woredas in northwest and Somalia, respectively (37, 41). Children who are not immunized are more likely to experience recurrent illnesses with diseases that can be prevented with vaccination, such as diarrhea and respiratory infections, which are known to deplete the body of nutrients (37).

Children who were not exclusively breastfed had odds of acute malnutrition that were roughly 2.0 times higher than those who were. This result was consistent with research from Bangladesh, Palestine, and Ethiopia that were published (32, 35, 38, 42, 43). Due to differences in sample size and study design, a Malaysian study that attempted to identify a link between the length of exclusive breastfeeding and children’s nutritional status was unable to do so (34).

Children from households without latrines were twice more likely to be odd or severely malnourished than their counterparts from households with latrines. One justification that may be made is that the use of latrines may imply a number of health benefits related to hygiene. This outcome was consistent with studies conducted in the Oromia region in western Ethiopia and the Shashogo woreda in southern Ethiopia (32, 36).

Children from households with an income of less 1,500 birr were substantially more likely to suffer from acute malnutrition. This is consistent with research from Bangladesh, India, Shinileworeda, the Ethiopian Somali region, Machakel woreda, and Northwest Ethiopia (9, 37, 38, 41). Even though income may not be a particularly accurate indicator of a household’s financial situation, increased household income could lower the incidence of acute malnutrition by enabling the purchase of food and healthcare supplies.

According to research findings from the East and West Gojjam zones of Ethiopia’s Amhara region, the location of one’s dwelling was also determined to be a significant risk factor for acute malnutrition in the current study (44). This may be because moms in urban areas may quickly access nutrition-related information distributed through various mass media, which may make it easier for urban mothers to appropriately feed their children than their counterparts.

Even after accounting for other potential variables in the multivariable analysis, having more children than had ever been born in the home was revealed to be a significant predictor of acute malnutrition. Studies from Malaysia (34), Shashogo woreda southern Ethiopia (36) and Nigeria (45) all reported a similar conclusion. Families with more children face a significant load on their already limited financial and food resources, as well as a reduction in the quantity and quality of care they may receive (45).

The incidence of acute malnutrition in children was significantly correlated with daily household water consumption of 50 liters. This is in line with the report from Ethiopia’s Oromia regional state’s GutoGida district (46). According to research from Machakel Woreda in Northwest Ethiopia and Tahtay Adiyabo Woreda in Tigray Regional State, Ethiopia (42, 47) water purification by straining using cloths was substantially linked to acute malnutrition.

The results of the current study also indicated that newborns of mothers who did not attend medical facilities during ANC follow-up were at risk for acute malnutrition. This is consistent with research findings from Oromia, Shinile, and Kemba woredas in southern Ethiopia (32, 35, 37) as well as the Ethiopian Somali region’s Shinile woreda. But according to research results from the Shashogo woreda in southern Ethiopia (36), maternal ANC follow-up was not significantly related. These discrepancies could result from different ANC service characteristics.

Compared to children whose weight was measured at birth, those whose weight was not recorded at delivery had a 0.3 lower risk of developing acute malnutrition. This is in line with research from Ethiopia’s Oromia regional state’s Guto Gida district (43). This could be as a result of poor health service standards and irregular ANC follow-up.

Compared to children born from a parent’s intended pregnancy, those born from an unexpected pregnancy were more likely to suffer from acute malnutrition. In contrast, a research from the Shashogo woreda in southern Ethiopia found the opposite (39). Variations in the research area and sociodemographic traits may be to blame for these discrepancies.

The results of this study demonstrated that aging is a risk factor for developing acute malnutrition. This may be because supplementary meals were introduced too late, there was insufficient food intake in terms of quantity and/or quality throughout the weaning and older ages, or dangerous supplemental foods were introduced, which could raise the risk of contracting diseases. This result is in line with a study carried out in southern Ethiopia (35).

Consumption of other fruits and vegetables, as well as dairy products, was inadequate, and they were important risk factors for acute malnutrition. Similar results were reported from the GutoGida district in Ethiopia’s Oromia regional state (43).

In the last 4 weeks, children from households who were unable to afford their favored foods had 3.2 times the likelihood of developing acute malnutrition. This is comparable to a Malaysian study that indicated that households with food insecurity at the level of child hunger were 16 times more likely to have malnourished children (34).

## 5. Strength and limitation

With a sound design and a sufficient sample size, the study was carried out among the most vulnerable kids in Gambella, a developing region of Ethiopia. The study may have been biased in the selection of cases because controls and cases were chosen from the same facility, however controls were deliberately chosen. It is also acknowledged that memory of information still has the potential to be biased; nevertheless, moms made an effort to get around this by responding by making associations with their own life events. The appropriate temporal association between exposure and disease cannot be established because of the inherent characteristics of the study design.

## 6. Conclusion

This study has identified a number of risk variables that may help public health professionals in their work when they engage with all relevant organizations. Therefore, household income of <=1,500 Birr, mothers’ unemployment, location, age of the child, total number of children ever born, daily *per capita* household consumption of water below 50 l, method of water treatment, lack of access to a household latrine, non-exclusive breastfeeding, minimum dietary diversity, lack of weight measurement at birth, unplanned pregnancy, non-immunization, failure to take vitamin A supplementation, and childhood illnesses (diarrhea, fever, and respiratory diseases),pre-pregnancy weight in the last pregnancy of <45 kg, absence of ANC follow up, inadequate intake dairy products and other fruits and vegetables, and inability to eat preferred foods in the past 4 weeks due to lack of resources were associated with acute malnutrition.

### 6.1. Recommendation

Health intervention initiatives should be conducted in an organized manner at all levels of the system in light of this discovery, which has significant program implications.

Regional and district health should aim to improve programs for food security, nutritional diversity, individual living standards, and maternity and child health care.

Health care providers including health extension workers should provide interventions for child hood illnesses such as diarrhea and pneumonia. Offer effective Educational programs to promote better child immunization, child feeding habits, better sanitation and hygiene, ANC follow-up, and family size.

Local non-governmental organizations (NGOs) working on nutrition programs should cooperate with district and regional health offices, as well as other stakeholders, to provide trainings to improve their living standards and close gaps caused by food insecurity. Future researches are recommended to conduct community based longitudinal study integrating with qualitative study design incorporating socio cultural determinants.

## Data availability statement

The original contributions presented in the study are included in the article/supplementary material, further inquiries can be directed to the corresponding author.

## Ethics statement

The Institutional Review Board of Mekelle University’s College of Health Sciences granted ethical approval. The Gambella Regional Health Bureau and the Gambella Teachers’ Education and Health Science College provided letters of support for the study. After outlining the study’s goals, methods, and participants’ freedom to opt out at any point during the interview, informed consent was gained from the subjects’ parents or legal guardians. Since there was no procedure that would have an impact on the study subject and the data would only be used for research, the Research and Ethical Review Committee also approved its ethical concerns. Information about patients and the hospital was kept private.

## Author contributions

LK and AG carried out all the conception and designing of the study, data collection, performed statistical analysis, wrote the final report, and reviewed and edited the final draft of the manuscript. All authors have read and approved the final manuscript.

## Conflict of interest

The authors declare that the research was conducted in the absence of any commercial or financial relationships that could be construed as a potential conflict of interest.

## Publisher’s note

All claims expressed in this article are solely those of the authors and do not necessarily represent those of their affiliated organizations, or those of the publisher, the editors and the reviewers. Any product that may be evaluated in this article, or claim that may be made by its manufacturer, is not guaranteed or endorsed by the publisher.

## Figures and Tables

**Table:** 

AIDS	Acquired Immunodeficiency Syndrome
ANC	Antenatal Care
BCG	Bacillus Calmette–Guérin
CMAM	Community based Management of Acute Malnutrition
DALY	Disability Adjusted Life Years Lost
DDS	Dietary Diversity Score
EDHS	Ethiopian Demographics and Health Survey
FMoH	Federal Ministry of Health
GTP	Gross Transformation Plan
H/A	Height for Age
HH	Household
HIV	Human Immuno-deficiency Virus
HFIAS	Household Food Insecurity Access Scale
HSDP	Health Sector Development Program
IPW	Inpatient Pediatric Ward
LMIC	Low and Middle-Income Countries
MAM	Moderate Acute Malnutrition
MUAC	Mid Upper Arm Circumference
POD	Pediatric Out Department
SAM	Severe Acute Malnutrition
SSA	Sub-Saharan Africa
UNICEF	United Nation Children’s Fund
UN	United Nations
WHZ	Weight for Height *Z*-score
WHO	World Health Organization
